# Foreign

**Published:** 1841-12-18

**Authors:** 


### FOREIGN.

*Case of accidental rupture of the Uterus, with escape of one or more fœtuses into the abdominal cavity.* By Henry R. Randell, M.R.C.S.—Mrs. ——, of this village (Acle,) ætatis 35, of pale aspect, but plump appearance, sent for me in the evening of the 3d of April, 1841, in consequence of her having been attacked during the day with pain in the lower and fore part of the abdomen, recurring at intervals of about a quarter of an hour. She stated that she believed herself to be about five months advanced in pregnancy, and had felt the motions of the fœtus about three weeks previously. She had passed water repeatedly since the commencement of pain, but there had been no escape of any other fluid from the vagina. The pulse was 92; tongue a little white, and she was thirsty. The bowels had not been relieved for three days, and she was naturally very costive. Whilst sitting by her bed-side she experienced a recurrence of pain; and placing my hand upon the abdomen, I felt immediately a decided uterine contraction.

There was a good deal of tenderness generally about the whole abdominal surface, which she said she had felt for some time. I remarked to her also the extreme enlargement of it for a person no further advanced in pregnancy than she had stated herself to be; and in reply she observed, that she was not exactly certain, for she had been taken very unwell about six weeks previously, and had been once slightly so since that period, and ever since she had felt altogeher very strangely at t'mes. She was certain, however, she had not felt the child until within the last three weeks, and then but very faintly. Considering it probable that premature labour was about to commence, I merely ordered her to keep quiet in bed, and to take an ounce of castor oil immediately; and if it did not relieve the bowels by the morning, to repeat the dose, and likewise to foment the abdomen by warm moist flannels, and to acquaint me in the event of a recurrence of pain.

4th.—She passed a restless night until near morning, when, after copious relief from the bowels, the pain left her, and she fell asleep for a few hours. There was, however, some little feverish excitement remaining, to remove which I ordered two scruples of the citras potassæ every four hours, and directed her to take only broth, tea, and gruel, in the way of diet, &c.

Towards evening she again complained of pain, but it was of a different kind, and occupied only the left hypochondrium, and was of a sharp stitchy character, more like that arising from intercostal rheumatism. The quiet state of the pulse, and abatement of the feverish state in which I found her in the morning, convinced me it was not inflammatory but rheumatic; and, upon inquiry, I found that the door, which is close to the bed side, was kept open during the time the attendant had been fomenting the abdomen on the previous night: the weather also being very cold, and no fire in the room, it left no doubt in my own mind that it arose entirely from this cause: I therefore merely ordered her an anodyne draught to be taken immediately, and the side rubbed twice a day with this embrocation—

Liquor Ammon. Fort., Tinct. Opii, aa. ʒij.;

Lin. Camph. c. ℥j. ft. embrocatio.

On the 5th she was better, had passed a good night, and was free from pain. I therefore merely ordered her to continue the powders and embrocation.

In the evening, however, she again sent for me in consequence of an increase of pain, which had resumed the periodical character it first had; and therefore, to satisfy my mind fully, I proposed an examination per vaginam, to which she assented. On the closest investigation, however, I found not the slightest dilation, or discharge; but the os tincæ appeared as small and as rigidly contracted as in the virgin state; I therefore simply ordered her to repeat the anodyne draught as before, to use poppy fomentations, and, as the pain was seated completely on the left side, afterwards to apply a blister.

On the 6th she was much better. I merely continued the powders. The blister had entirely removed the pain. The bowels, however, had not been relieved since the 4th; I therefore requested her to repeat the castor oil.

From this time she daily improved until the 10th, when, in the night, she was suddenly seized with hæmorrhage from the uterus, accompanied with occasional small grinding pains, which continued from 2 o'clock, A.M. until 8 A.M., when both appeared as suddenly to cease. She stated, however, that, although she felt in every respect better, she had been very faint during the night; and, notwithstanding this feeling had in a great degree gone off, yet she still felt very "oddly." This determined me to make another very careful external examination, for I entertained some suspicion, from the altogether unusual character of her symptoms, that there was something extraordinary in the case, which I could not satisfactorily account for. On doing so I was instantly and very forcibly struck with the very plain manner in which I could now feel the fœtus, its knees, elbows, &c. and below it apparently the enlarged uterus very distinctly. The whole abdomen, too, appeared enlarged since my first examination, and the child appeared to lie transversely across it, principally above the umbilicus. I merely ordered her to keep quiet in bed until I should see her again, to live on a slight spare diet, and take a sixth part of the following mixture every four hours:—

Potass. Nitrat. ʒij.; Tinct. Opii, gr. xxx.;
Infus. Rosar. ℥viij. ft. mistura.

In the evening of this day the pain abated, and she felt altogether better.

11th.—Has passed a good night, and had no return of pain or hæmorrhage, I now made another careful examination both externally and internally, and the result was that I became convinced, from the very plain and unusually distinct manner in which I could define the head, limbs, and trunk, of the fœtus, its ribs, &c. that it was a case of extra uterine fœtation. The os tincæ was still closed, and its neck appeared so much contracted as to render it extremely tuberose to the touch. All hæmorrhage had ceased as well as pain. The extraordinary nature of the case rendered it desirable that my own opinion should be confirmed by another's, and I therefore at once proposed a consultation. Dr. Lubbock was soon after called in, and having instituted a very minute and careful examination, he came to the same conclusion as myself, that it was undoubtedly a case of the nature I have described, and wished that exactly the same plan of treatment should be pursued, and the patient for the present left as much as possible to nature.

On the 12th there was a slight return of hæmorrhage, but no change in any other respect.

On the 13th she was much the same, and I therefore ordered ʒij. Tinct. kino, to be added to the mixture, and napkins soaked in equal parts of vinegar and water to be worn.

On the 14th the discharge had ceased, and only a small quantity of watery fluid escaped at the time of passing the urine; she therefore discontinued the napkins, and simply went on with the mixture. I had all along ordered her to be kept quite cool, and to have plenty of fresh air, to wear a loose dress, and in the daytime to repose on the outside of the bed.

She continued in this way, without any material alteration in her feelings, until the 20th, with the exception of all loss of feeling respecting the fœtus, since the last attack of hæmorrhage, and that the abdomen appeared to be more relaxed and flabby. The fœtus, too, which, if possible, was to be more distinctly felt than ever, she remarked, appeared to settle or gravitate to the most depending part, according to whichever side she rested upon; although its principal situation was still on the right side of the umbilicus, and a little above it. On the morning of this day, however (20th,) she had been attacked with small grinding pains, and had passed a few coagula, mixed with what appeared to be portions of deciduous membrane. I therefore made an examination per vaginam, and for the first time felt the os tincæ relaxed and dilated to the extent of a crown-piece, and its mouth occupied by a coagulum. On removing this, and introducing the index within its cavity, I felt, although scarcely within touch, something like the head of a fœtus. I therefore directed her to keep very quiet, to take only broth and gruel alternately, in order to support her strength, and to acquaint me as soon as the pains begin to increase. I heard no more from her till six in the evening, when, on my return from visiting my other patients, I found they had twice sent for me.

On examination at this time I found the os uteri two-thirds dilated, the fœtal head presenting, and just dipping below the superior margin of the pelvis, and that there had been a constant draining of arterial coloured blood during the day. The pulse, however, was good, she had taken a good deal of nourishment, and had not once complained of feeling faint. As the pains appeared tolerably efficient, I suffered her to go on without rendering her any more than the ordinary assistance; but after waiting three-quarters of an hour, finding the head made no progress, and that the pains also began to flag, I gently introduced the vectis, and speedily extracted the fœtus, which appeared to have attained the age of seven months. But little contraction of the uterus followed, although I gave her (as is my usual custom) 35 drops of laudanum, and half an ounce of brandy, in a wine glassful of cold or tepid water, and made use of pressure and friction with my hand on the abdomen for twenty minutes or longer. On doing so, however, I still felt the fœtus in the abdomen as plainly as ever, only that it now appeared to have sunk a little lower down; and thinking it just possible that the parietes of the uterus might be extraordinarily thin in this case, so as to deceive me as to its being extra-uterine (although I believed I could still plainly distinguish the uterus by itself in the hypogastric region, only more contracted than before,) I determined, as there were no urgent symptoms, to wait a little, previous to my attempting to extract the placenta, as is usual with me at the expiration of this portion of time from delivery. No flooding or any other untoward symptom succeeding, I waited for an hour and three-quarters, when, for the first time since the birth of the fœtus, she suffered a slight pain; this was soon followed by others, and by gentle flooding. I immediately introduced my fingers as high as I could reach, and felt nothing but the placenta. I then gently introduced the left hand into the uterus, and still no fœtus was to be felt.

Having satisfied myself, therefore, that the other child was not in the cavity of the uterus, I proceeded at once to extract the placenta, which I found partially adherent to the fundus uteri; and on separating it carefully with my nails throughout its whole extent, I found a portion of it elongated, and stretched toward the upper and back part of the left side of the uterus. On tracing this elongated portion very carefully, I found that it perforated the uterus at this part, by means of what appeared to my touch a somewhat narrow irregularly oval sort of opening, the edges of which felt contracted and tense, and which was barely sufficient to admit the index more than half way, and beyond which I could feel nothing but what imagined to be softened placenta. I therefore detached this elongated portion of placenta, which readily gave way on slight stretching, and removed the whole. The contraction of the uterus followed the immediate withdrawal of the hand, and consequently there was no further hæmorrhage.

I immediately placed my hand upon the naked abdomen, and could now distinctly feel the empty and contracted uterus; the fœtus before mentioned in nearly the same situation; and above the situation of the iliac fossa in the left side, another substance, which gave the idea, from its irregularity, &c. of another fœtus; but this, of course, was doubtful, as I could not clearly define it. The case, however, was now perfectly clear to my own mind, viz. that she had sustained, by some means or other, a rupture of the uterus, and that the fœtus or fœtuses had escaped from the uterus into the abdominal cavity, whilst one, and most likely that which happened to be most inferiorly situated, had remained. Or it might be that the retained one had pursued its proper course, whilst the other or others had escaped from the fallopian tube at the first into the abdomen, if at least such a case be possible. Upon inquiry, however, subsequently, I learnt that about six weeks prior to this period, she was one day in the act of pumping from a pump, the handle of which was suspended higher than usual, and that consequently she had been obliged to use considerable exertion in order to raise the water; and, whilst doing so, she experienced a very strange sensation in her abdomen, accompanied with fainting, which obliged her instantly to come in and lie down on her bed. Very soon after she was taken unwell, with a good deal of pain and soreness, and that these symptoms continued with very little variation for nearly a week, and obliged her, from the excessive feeling of weakness they induced, to keep her bed nearly all that time. After keeping perfectly still, however, for the period I have mentioned, and taking a little castor oil occasionally, she began to mend, although she had not felt so strong ever since as she had done before, and fouud herself incapable of the least exertion. Such was the account she gave me.

Having gently supported the abdomen with a broad bandage, I directed her to be placed in bed, and the most perfect quietude enjoined. At the end of an hour I again visited her, and found her as comfortable as could be expected. No flooding had supervened, there had been but very slight pain, and she seemed disposed to be affected by the opiate. I therefore left her for the night, desiring that I might immediately be sent for if any change arose in the symptoms.

21st.—I visited her at half-past six, and found she had slept only half an hour. She had felt but very little pain, but complained of very great exhaustion, and cold clammy perspirations. Pulse 132, and feeble: no hæmorrhage, however, but a very copious discharge of highly offensive watery fluid. There was but very little tenderness of the abdomen, and, on examination the uterus could be felt firmly contracted, just above the pubes, and the two other fœtuses as plainly as before. No urine had been passed. I ordered her to take a fourth part of the following mixture every four hours.

Ammon. Subc. ʒij.; Tinct. Cinch. Co., Tinct. Hyoscy. aa. ʒij.; Mist: Camph. ℥vj. ft. mist.

At the end of a week from delivery I again carefully examined the abdomen externally, and now as plainly as ever felt the fœtus in the hypogastric region: the presence of a second fœtus was, however, no longer clearly to be detected. The lochial discharge, though irregular, still continued, but was less fœtid, and the general health was rapidly improving.

May 27th.—From the last date to the present period she had been progressively improving and gaining strength.

August 7th. Up to the present period she has contiuued as well as might be expected. She still retains a more than usually blanched countenance, and is incapable of much exertion, although she walks out a short distance whenever the state of the weather and roads permit. The catamenia have returned and are regular.—*London Med. Gaz*

---

*Action of Hydrocyanic Acid, etc. upon the Eye.* By A. TURNBULL, M. D.—It is a well-known fact that the eyes of those who have been destroyed by hydrocyanic acid for a length of time after death show none of the usual symptoms of dimness. On the contrary, the eye is clear, and the pupil much dilated. This satisfied me that the acid exerted a specific action upon the eye, which might be made available as a medical agent for relieving many of the diseases to which that organ is so subject.

My first experiment was undertaken in 1837, with the diluted acid, by dipping a sponge into it, and rubbing it upon the forehead for the space of a few minutes, which gave the skin a very red appearance; but the patient experienced not the least sense of heat, and the pupil was slightly dilated. I continued to use this with very beneficial effects in incipient cataract, opacities of the cornea, inflammation, amaurosis, iritis, &c. Of late I have substituted the vapour of the concentrated acid to the eye with much more decided effect, and without the slightest danger. The plan I generally adopt, is to put into an ounce-phial a drachm of the acid, and hold it in close contact with the eye, the eyelid being open, for the space of about half a minute, or until such time as the patient feels a little warmth, or the person holding the phial sees the pupil greatly dilated, and the vessels of the eye injected with blood, which is the invariable effect of the application of the acid. The patient is not sensible of pain from this peculiar state being induced, which appears to me to result from the powerfully sedative influence of the acid, thereby showing that two opposite powers—to wit, stimulating and sedative—are exerted at the same time; and thereby the uneasiness arising generally from a stimulant alone is prevented. Its great power in removing these diseases chiefly arises from the two powers being so blended, and thus enabling the eye to bear a sufficient stimulating action without injury. The person who holds the acid to the eye should be careful not to allow the patient to smell it.

The essential oil of bitter almonds I use for the same diseases. I put two drachms of water to two drachms of the oil, in an ounce-phial, and hold it in the same way to the eye as the acid; but its effects are not precisely alike. The feeling induced by the oil is soothing, and generally relieves all sense of pain, even of tic douloureux, without sensibly dilating the pupil, or causing much redness of the eye. I find it very useful in taking away the heat occasioned by the hydrocyanic acid.—*London Medical Gazette.*

---

*Extra-Uterine Fœtation. Removal of the Fœtus through the Vagina.* By M. Voillemier.—Baudelocque first conceived the idea of delivering women affected with extra-uterine conceptions, through the vagina. Since his time the operation has been often performed; and sometimes the mother, sometimes the child, and sometimes both, have been saved. Whenever it can be performed, the operation is preferable to gastrotomy; but should it be resorted to when the fœtus is certainly dead? This is a question still to be resolved; for so many cases have been seen in which fœtuses, having become congested, have given no trouble for several years, and so many in which women have died of operations, that in general these cases have been left to nature. Dubois and Boyer thus left a case, of which the remarkable history was given by M. Thivet (Bull. de la Soc. Anatomique, 1839): the fœtus remained for eighteen years in its mother's peritoneal cavity, and she died of a strangulated umbilical hernia independent of it. In other cases, after an uncertain length of time, a process is set up by which the fœtus or its *debris* are discharged either by the rectum or by the vagina, and then the cure may follow; but in yet other cases the cyst opens into the peritoneum, and then a peritonitis destroys the patient; or else fistulous abscesses are formed in several directions at once, into the intestine, the urinary and genital organs, and the abdominal walls; and these are followed by death, or very slowly by recovery. Such accidents might have been feared in the following case by M. Voillemier:—

The patient having escaped the first dangers which attend every extra uterine pregnancy, and which seemed still more imminent on the day when labour pains came on, thought herself suddenly delivered: the pains ceased, the abdomen diminished in size, and she regained her health. But after a year, without any known cause, she had dull pains in the hypogastrium; and, her health being again deranged she came into the Clinical Hospital on the 18th of August, 1838, being then forty-one years old. After making the necessary inquiries of preceding history, and an attentive examination of the abdomen, a large fœtus was discovered. Above the upper aperture of the pelvis on the right side, there was a round solid tumour formed by the head; higher up, and towards the left and forepart, there was a flat surface, with some irregularities, which it was thought was formed by the vertebræ; and more to the right, and below the ribs, there was another tumor presenting several projections, but nothing very distinct. The fœtus, therefore, appeared to be placed in nearly the first position. By the side of the vagina, fixed in the upper strait, a round hard tumor, evidently formed by the head, was felt: it was covered by only a soft and thin envelope, and the saggital suture could easily be felt. The uterus was thrown behind the horizontal ramus of the right os pubis, and had undergone no other change than that of position.

On the 21st of August, M. Dubois performed the operation. After having introduced a short and large speculum into the vagina, he made along the tumour a transverse incision, which penetrated to the bones of the head; but no liquid following, and finding no separation of the divided tissues, he feared there might be adhesion of the head of the fœtus to the lower part of the cyst, and he had the patient put to bed again. On the same evening a fœtid puriform discharge through the vagina took place; on the next day there was a little fever; on the 24th, nausea, and great tenderness on pressure of the abdomen. Leeches, Seidlitz water, &c. were ordered. On the 26th there was more pain; the bones of the head were felt shifting one upon the other. On the 27th several portions of them were extracted: the discharge was very abundant, but was not now fœtid. On the 6th of September, the bones were extracted with difficulty. Two hours after there were violent rigors; but the fears which they excited were soon relieved, and next day the patient got up. Slowly walking and injections promoted the descent and discharge of the other bones; but the sac had to be examined again to extract some of the long ones. On the 28th of September the patient went out completely cured. —*London Medical Gazette, from L'Examinateur,* Juin 27, 1841.

*Case of Scirrhous Stricture of the Œsophagus, with Clinical Remarks at Charing Cross Hospital.* By Dr. CHOWNE.—James Upcott, aged 61, light hair, eyes, and complexion, by trade a tailor. He was from sixteen years of age to twenty-nine in the navy, and during that time lived and drank freely. Since he was twenty-nine years of age he has been a sober liver. He was admitted into hospital under Dr. Chowne on the 6th of August, 1841; he had been twice previously in the institution, in each instance for pain in the left hypochondrium; and in both, after the use of mercurial alteratives and aperient medicines, went out well. There does not appear to be any obvious connection between these illnesses and the disease for which he was last admitted.

In April last he began, without any particular pain or disorder, to be sick after taking food; this continued, and he obtained admission to the hospital on the 6th of August. At that time he incessantly threw up every thing he took; but the act of doing so was neither preceded, accompanied, nor followed by pain. On examining the body no fulness or tumour was perceptible at the scrobiculus cordis, or elsewhere, nor did pressure give pain. The pulse was usually about eighty-six, small and regular; skin cool and dry; tongue moderately clean; bowels very inactive, but the alvine secretions natural. This was his general state. The sickness came on *immediately* after swallowing, and resisted every means used to prevent it; no applications, internal or external, had any permanently good effect; and he gradually sunk, and died the 5th of September, thirty days after his admission. He was without pain to the last.

*After-death Appearances.*—The thorax presented nothing abnormal, except adhesions between the pleura pulmonalis and pleura costalis; the liver was rather indurated, but not particularly altered in size or structure; the spleen about a quarter larger than common, more firm and more florid, fleshy, and at the same time granulated in appearance; the blood which oozed from it was of a claret colour. The pancreas appeared to be much wasted, and was so thin as not to be readily recognized; its colour was darker, and more livid than common. The œsophagus, from its commencement to the diseased portion, which was involved with the cardiac orifice of the stomach, quite natural in texture and in size. Mucous membrane not ulcerated. The termination of the œsophagus and the cardiac orifice were in a scirrhous state. The scirrhous mass was about the size of a pigeon's egg, firm under the scalpel; the cut surfaces greyish white and opake, but containing small circumscribed deposits of a darker hue, and rather transparent; stomach healthy, but small. Nothing remarkable in the other portion of the intestinal canal. The scirrhous portion was irregular in shape, both externally and internally; the cardiac aperture was all but closed, as it would with difficulty admit a crowquill.

Dr. Chowne observed, that two circumstances had tended to embarrass the diagnosis in this case. The first of these consisted in the fact that scirrhus, in the situation which it occupied in the present instance, frequently gave the patient the sensation of positive dysphagia, and food was so immediately rejected as to lead the patient to feel that it had not descended; but, in the present case, the patient had the sensation of having completely swallowed the mouthfuls that were taken, although extremely little of any thing, even of fluid, could possibly have passed into the stomach.

Secondly. Scirrhus generally produced pain extending from the region of the scrobiculus cordis to the back. In Upcott's case there was no pain. There were, nevertheless, strong reasons for considering that the obstruction was at the cardiac orifice; firstly, the vomiting occurred immediately after deglutition; in scirrhus of the stomach itself, and of the pylorus, there was generally an interval of about a quarter of an hour; secondly, the food was thrown up in the state in which it had been swallowed, and mixed with mucus only; in scirrhus of the body of the stomach the food was often mixed with a blackish fluid, and where the disease was situated in the pylorus, the food was thrown up more altered by the process of digestion, and in larger quantity.

Thirdly. There was no tumour perceptible in the epigastrium; in scirrhus of the pylorus a tumour was generally perceived, and in scirrhus of the stomach there was also sometimes a tumour perceptible through the abdominal parietes. The œsophagus being found of its natural calibre at all parts above the disease, was in accordance with the circumstances of the case. Great diversity was seen in different cases in the degree of tolerance exercised by the œsophagus; in some there was a great degree of tolerance and consequent dilatation. A case was on record in which there had existed dysphagia from disease of the cardia orifice of the stomach; the œsophagus became distended into a sort of pouch from two inches below the pharynx to the diseased part, capable of containing two quarts. In a patient under his own (Dr. Chowne's) care some time since with stricture in the lower part of the œsophagus, solids taken at intervals and in small quantities were retained for a considerable time, but were finally thrown up in masses described "like penny-pieces piled one upon another." In this case the œsophagus was somewhat larger than common; in the case of Upcott, the tolerance was extremely limited, and the œsophagus retained its natural size. Immediately that the sensation of swallowing terminated, that of sickness supervened, and what little had been taken was immediately ejected. The unfortunate patient took spoonful after spoonful of broth, egg, wine, and other kinds of nourishment, with the fore-knowledge that it would not be retained, and with a vessel by his side to receive it; his taking nourishment by the mouth, indeed, was an alternation of deglutition and vomiting; the vomiting was not only unattended by pain, but was also unattended by loss of appetite; indeed, appetite was extraordinarily, if not morbidly, strong: so great was the yearning for food, that, to use the poor man's own description, "if he had not had a supply of food at hand, he could scarcely have refrained from taking again that which had been returned." In speaking of the lining membrane of the œsophagus, it was remarked that there was no morbid change. It was very common, however, to find that ulceration had taken place in the vicinity of the obstruction; this was found in mere strictures of the œsophagus, as well as of other parts. When it occurred in the œsophagus, it was sometimes attributed to the vomiting, although there might be great reason to suspect a predisposition of a kindred character to that in its neighbourhood. In the case of Upcott, the scirrhus itself had not proceeded to ulceration, which might partly explain the healthy state of the upper part of the canal. To this might also be attribtued the absence of pain in deglutition. Swallowing, or rather the attempt to swallow, where there was scirrhus of the æsophagus, was usually a painful effort, particularly when spirit was mixed with the aliment; but Upcott had no pain even from the brandy and water which he sometimes took in preference to wine. His mind was perfectly tranquil, and he retained his mental faculties, as was common in such cases, to the last.

*London Lancet.*

---

*On the Internal use of the Nitrate of Silver in Inflammation of the Intestines.* By John James Macgreggor, M. D., Physician to the South-Eastern Dispensary, Dublin, &c.—L. M., a lady, aged twenty-three, in a most precarious state of health for some years, and constantly requiring medical aid, the uterine and digestive functions much deranged, whole appearance strongly indicative of the existence of latent tubercle, although no pulmonary symptoms had as yet set in, was attacked with diarrhœa in the month of April, 1840, while on a visit to the country. The bowel complaint continued with slight intermission till the month of June, when, in the middle of the night, I was sent for, by her medical attendant's advice, to see her. On this occasion she was suddenly seized with a violent spasmodic attack, which, however, soon subsided. My attention was directed to the state of her bowels. I was informed that her stools averaged from six to seven in the course of twenty-four hours: upon examination they proved to be highly fœtid and of a slimy consistence, more or less mixed with scybalæ. She complained of much pain immediately before and after each stool, and had excessive tenesmus and occasional prolapsus ani. The abdomen was distended and tympanitic, and intolerable pain was felt upon the most moderate pressure of the hand, particularly over the right iliac region. Her pulse varied from 112 to 120, was sharp, but easily compressed; skin hot; great thirst; inappetence; she was extremely emaciated and excessively weak; her face was blanched, and highly swollen about the eyelids; she had no cough, and auscultation elicited no morbid sign, except severe palpitation of the heart from nervous irritation.

Her gums were slightly affected from alterative doses she had been taking of hydrargyrum cum cretâ and Dover's powder before I saw her. We ordered four leeches to be applied over the right iliac region where she complained of much pain, and an enema of chicken broth was administered, from which she obtained temporary relief, but in a few hours the diarrhœa and pain returned. The acetate of lead had been tried in an enema without any good result, the diarrhœa soon recurring with increased violence, as if the sudden arrest of the secretion, without altering the seat of inflammation, had only served to increase it. A grain and a half of the nitrate of silver in two ounces of distilled water, with half a drachm of the acetous tincture of opium, was given in the form of enema, and repeated occasionally with marked benefit. The stools were reduced to four in the course of the four-and-twenty hours, but the pain still continued in the right iliac region. A consultation was proposed, and Sir Henry Marsh was called in to see the patient. It was resolved to continue the nitrate, and to give it in the form of pills, beginning with a grain dissolved in distilled water, and rubbed up with crumb of bread and one quarter of a grain of extract of opium in each pill, to be taken every sixth hour. The improvement which took place was gratifying: the stools were reduced to two in twenty-four hours, and the tenesmus was much lessened; blisters were applied to the abdomen, and the patient allowed to take broiled meat, wine, asses' milk with syrup of capillaire, &c. She was now permitted to leave her room, and take air and exercise in the garden, for two or three hours daily, in a Bath chair. The pain still continuing in the iliac region, an opiate plaster was spread over the abdomen. She slept tolerably well, and continued to improve under this treatment, without regaining much strength, or a return of her appetite, till the latter end of September. The diarrhœa had never returned, and there was little or no uneasiness upon pressing the abdomen. The stools had assumed their natural appearance and consistence. Having expressed a strong desire to be sent to the sea-side, her wishes were gratified, and during her residence there I visited her three or four times a week. Her spirits were more cheerful, but she was very weak and deadly pale. Towards the middle of October she complained of slight cough, and the palpitations became more distressing; pulse tolerable; sleeps well; strongly desirous of life; cough more distressing; effusion into the chest and cellular membrane; cough relieved by a blister. She was now removed into town at her own desire, when the effusion suddenly increased, and towards the end of October she expired without a struggle.

It is necessary to offer but a few observations on the above case. The diarrhœa, and other symptoms of diseased intestine, yielded almost immediately to the nitrate of silver, after having for two months resisted the whole catalogue of astringent medicines; and what is remarkable, from the month of June till October, when she died, never returned. She continued to take the pills to the last, without suffering any inconvenience from them. In this case of phthisis the late supervention of the pulmonary symptoms proves the correctness of the diagnosis at the period when they were entirely absent.

Case 2.—Patrick Rorke, aged twenty-one, tall and narrow-chested, high-shouldered, trade a shoemaker, applied for advice at the South-Eastern Dispensary in the month of July, 1840. He complained of an acute pain in his right side, which was increased upon taking a full inspiration; severe and hollow cough; copious yellow expectoration, streaked with blood; had been obliged to relinquish work from extreme weakness; had distressing dyspnœa and palpitation; lost flesh considerably; bad appetite; pulse 120, but compressible; most distressing diarrhœa and night-sweats.

I visited this patient at his own house, and discovered a large cavity beneath the clavicle in the upper lobe of the right lung. He could not bear the slightest pressure over the abdomen, and was on the night-chair every twenty minutes. A few leeches were ordered to the abdomen, with fomentations of poppy-heads and extract of belladonna. He was ordered to take a pill, composed of one grain of nitrate of silver, a quarter of a grain of extract of opium, and three grains of compound cinnamon-powder, every three hours. Upon the following day I found the stools had been reduced to four in the course of the twenty-four hours, and that he had slept better than for some weeks. He was allowed broiled mutton and porter: the night-sweats were also diminished. I saw him two or three times a week for nearly a month, during which time the diarrhœa had not returned, the stools being reduced to two in the four-and-twenty hours, without any pain. The cough remained stationary, and by my advice the patient went to the country, after which I never again heard of him.

In the above case no astringents nor opiates had been tried, the relief obtained from the nitrate of silver was immediate.

Case 3. *Ordinary Chronic Diarrhœa.*—Mary Brenan, aged forty, has had a most distressing diarrhœa for four months, for which she has been in hospital, and under the care of several medical men, without obtaining the slightest relief. She attributes the complaint to her having eat too much new vegetables. She was ordered to take the nitrate of silver, with watery extract of opium, in the proportions already mentioned. 12th August, she thought they disagreed with her, and increased the number of stools. I told her, however, to persevere, and on the following day she had only two stools. On the 14th the bowels remained unmoved. 15th, one stool; appetite much improved. I did not see her again for a week, when she was quite well.

*Case* 4.—May 5, 1840, John Boordman, aged sixty-four, florid complexion, by trade a weaver, applied for relief at St. Peter's Dispensary, stated, he had diarrhœa for the last eighteen months; going to stool at least a dozen times in the twenty-four hours. About a year and a half ago he had been operated upon by Mr. Colles, at Stephen's Hospital, for a tumour of the scalp, and was immediately after seized with a bowel complaint, which to the present period had resisted every means tried by medical men in England and Ireland; his trade sometines obliging him to visit the former country. He complained of no pain; and his stools were watery, and of a dark brown colour; very fœtid; no admixture of blood or scybalæ; no gastric symptoms; urine greatly diminished in quantity He was ordered,

> Nitrate of silver, gr. xij;
> Watery extract of opium, gr. iij;
> Extract of gentian, gr. xviij.

To be made into twelve pills; one to be taken after every liquid stool.

May 9th, he called on me, and stated he was much better; his stools were reduced from twelve to two daily; quantity of urine much increased. The pills were ordered to be continued.

15th, continues much better; stools two in the twenty-four hours, still dark-coloured and serous.

Aug. 10th, I did not see him again till this date, when he called on me, as I had desired him: remains perfectly well; has but one stool in the four-and-twenty hours.

*Observation.*—I was afraid of dropsy setting in in this case after the diarrhœa was checked, both from the long continuance and great quantity of the serous discharge, which was so suddenly arrested by the employment of the nitrate of silver, from a notion I had conceived that it was an effort of the constitution which might not permit any interference with impunity. The result, however, removed my apprehensions, especially as the kidneys resumed their natural function. I have no doubt that chronic inflammation of the bowels was the cause of the diarrhœa, both in this and the preceding case.

*Case.* 5--John Taafe, aged 27, by trade a sawyer, dark hair and sallow complexion, had several attacks of hæmoptysis, which were all relieved by venesection and acetate of lead; a well-marked cavity under left clavicle; distressing cough; emaciation, and profuse diarrhœa, attended with severe griping pains during stools, and mixed with scybalæ and clots of blood. This man had colica pictonum, from the use of the acetate of lead; which was removed by repeated doses of sulphate of magnesia, sulphuric acid, and distilled water. The diarrhœa, however, did not occur for two months afterwards.

On the 25th of August, 1840, he applied for relief; I found him in a most miserable state of debility, from pain and exhaustion; his stools amounting to thirty in the four and twenty hours; he was reduced to a skeleton, and every particle of food he swallowed compelled him instantly to go to stool. I ordered him a grain of the nitrate of silver, with half a grain of the watery extract of opium, to be taken every three hours, and a belladonna and opiate plaster over the abdomen. He was ordered chicken, and one part of lime-water to two of boiled asses' milk with syrup of capillaire.

On the 26th I found him much refreshed and in better spirits; he had only four stools in the twenty-four hours, and had got several hours of interrupted sleep.

He went on favorably, without any return of of the diarrhœa, till he omitted taking the pills for some days, when it again recurred, but not with such severity. I ordered him one-twelfth of a grain of strychnine, with half a grain of extract of opium; to be repeated after each stool.

On the 10th of September, no diarrhœa; was able to sit up in bed, and felt much lighter; but towards night he suddenly expired, from an attack of profuse hæmoptoe, brought on by a fit of coughing.

It would take up too much space, and would, in other respects, be useless to prolong this selection of cases: as those I have chosen, from several noted down, sufficiently attest the power of the nitrate of silver in the diarrhœa attendant upon inflammation of the mucous membrane of the intestines, whether of a secondary or primary nature. It is, however, in affording relief in the bowel complaint attendant on phthisis that I consider it particularly valuable, and in no single case that I have tried it was I disappointed. In a few the disease, after being at first benefitted, recurred again; but in almost all I found it to allay the pain and distressing tenesmus, and effectually arrest the alvine discharges, and thus afford days and weeks of ease and comfort to the suffering patient.

*London Lancet.*

We have used the nitrate of silver rather largely in the treatment of dysentery, and especially of the chronic variety of it. Although with other medicines it is by no means infallible, it is often most successful. One mode is to begin with a quarter of a grain three times daily and gradually increase it; say, doubling it every second day, until we reach a grain at a dose. If it should be necessary to persevere in it, the best plan would be to give the doses a little more frequently. Of course, constant attention should be paid to its action, and if the tongue become much more dry, or the stomach painful, the silver should be discontinued, or at least suspended.

---

*Exhibitions of small doses of Mercury to effect Ptyalism.* By John H. Tosswill, one of the Surgeons to the Leicester General Dispensary.—Having read a paper in the Lancet of last week by Mr. Clay, on the exhibition of small doses of mercury in effecting ptyalism, I have been induced to offer a few remarks, the result of my own observations in the application of the same remedial means. I do not think it necessary to enter fully into the symptoms and treatment of the cases which have come under my care, but merely to bring before your readers that part relating to the exhibition of mercury in small doses. My attention was first called to the subject by reading a communication by Dr. Law, in one of the Dublin medical journals about twelve months since, in which that gentleman spoke highly of the beneficial effects resulting from frequently repeated and minute doses of calomel in producing rapid constitutional effects. Possessing, at that time, numerous opportunities as visiting assistant to a large dispensary at Coventry, I determined on making a trial of the statements I had read. My first case was that of a young female, named Heywood, of delicate constitution, who applied for advice, labouring under an attack of pleurisy. Having bled her, and administered the usual medicines, she was ordered to keep in bed, and to have calomel, two grains; liquorice powder, q. s.: to be made into twenty-four pills: take one every hour, night and day. Having persevered in this treatment until she had taken two grains and a-half of mercury, she was obliged to discontinue their use, her mouth and gums being rendered painfully sore; so that, in this instance, in thirty hours, with confinement to the bed and after bleeding, my patient was sufficiently and decidedly under the influence of the treatmet. The next case, of which I possess notes, was one of quinsy. The patient, Mrs. Marr, ætat. 22, a dress-maker, of plethoric habit and sanguine temperament. Having applied leeches to the throat, and ordered other medicines, she had given calomel, two grains; sugar, q. s.: divide into twenty-four powders; take one every hour. In this instance, nearly five grains have been taken before any sign of ptyalism was produced, and, as may have been anticipated, after the lapse of forty-eight hours, the patient became weary of taking her powders with the regularity I had insisted on; yet, having taken five grains, her gums became affected, and I discontinued the use of the powders. More lately, as one of the surgeons to the large dispensary of this town, opportunities have occurred for my again recurring to this mode of treatment.

Thomas Kinsey, about 40, by trade a stocking-maker, of thin, spare habit and bilious temperament, applied to me for advice, suffering from acute inflammation of the liver. Having taken about eight ounces of blood from him, and administered a purgative mixture, he was ordered to have two grains of calomel, to be made into twenty-four pills, with liquorice powder, and to take one every hour. The patient attended regularly to my directions, and having taken five grains, the mercury was discontinued, the gums having become sore, and the effect of the medicine completely established, he was subsequently ordered a blister over the region of the liver, and rapidly became convalescent.

The only other instance in which I have lately had an opportunity of making trial of small doses of mercury, was in the case of an excavator, named Moore. The patient was strong and very muscular; he complained of acute pain in the region of the right kidney, increased on pressure, with rheumatic pains in the joints. Ordered to be cupped on the loins to six ounces; and, in addition to other remedies, to have three grains of calomel made into thirty-six pills, with liquorice powder; to take one every hour. On visiting my patient the following day, I was surprised to find that two grains and a sixth of calomel, or twenty-six pills, which he had taken, had produced decided ptyalism; he was therefore ordered to discontinue their further use. From the limited number of cases which I have treated in this way, I should have forborne making any observations on the subject in a public journal, had I not thought them confirming Mr. Clay's views, and as such I beg to offer them, coinciding as I do with that gentleman, and feeling convinced that small doses of mercury, frequently repeated, are more rapidly efficacious than large ones, administered at long intervals.

*Lancet.*

We have not tried this practice precisely, but have at times adopted one very similar—giving an eighth of a grain of calomel every two hours. We believe that a much smaller dose than that usually prescribed would be sufficient to excite ptyalism.

